# Patient-reported cosmetic satisfaction and the long-term association with quality of life in irradiated breast cancer patients

**DOI:** 10.1007/s10549-019-05470-y

**Published:** 2019-10-24

**Authors:** M. C. T. Batenburg, M. L. Gregorowitsch, W. Maarse, A. Witkamp, D. A. Young-Afat, A. Braakenburg, A. Doeksen, T. van Dalen, M. Sier, E. J. P. Schoenmaeckers, C. H. van Gils, H. J. G. D. van den Bongard, H. M. Verkooijen, the UMBRELLA study group

**Affiliations:** 1 Department of Radiation Oncology, University Medical Center (UMC) Utrecht, Heidelberglaan 100, 3584 CX Utrecht, The Netherlands; 2 Department of Plastic Surgery, University Medical Center Utrecht, Utrecht, The Netherlands; 3 Department of Surgery, University Medical Center Utrecht, Utrecht, The Netherlands; 4 Department of Plastic, Reconstructive and Hand Surgery, Amsterdam University Medical Center, Amsterdam, The Netherlands; 5 Department of Plastic Surgery, St. Antonius Ziekenhuis, Nieuwegein, The Netherlands; 6 Department of Surgery, St. Antonius Ziekenhuis, Nieuwegein, The Netherlands; 7 Department of Surgery, Diakonessenhuis, Utrecht, The Netherlands; 8 Department of Surgery, Ziekenhuis Rivierenland, Tiel, The Netherlands; 9 Department of Surgery, Meander Medisch Centrum, Amersfoort, The Netherlands; 10 Department of Clinical Epidemiology, Julius Center for Health Sciences and Primary Care, University Medical Center Utrecht, Utrecht University, Utrecht, The Netherlands; 11 Imaging Division, University Medical Center Utrecht, Utrecht, The Netherlands; 12 Utrecht University, Utrecht, The Netherlands

**Keywords:** Breast cancer, Cosmetic outcome, Radiation therapy, Quality of life, Longitudinal

## Abstract

**Purpose:**

To evaluate patient-reported cosmetic satisfaction in women treated with radiation therapy for breast cancer and to determine the association between dissatisfaction and quality of life (QoL) and depression.

**Methods:**

Within the prospective UMBRELLA breast cancer cohort, all patients ≥ 1 year after breast conserving treatment or mastectomy with immediate reconstruction were selected. Self-reported cosmetic satisfaction was measured on a 5-point Likert scale. QoL, social functioning, and emotional functioning were measured using EORTC QLQ-C30 and BR23 at 1, 2, and 3 years after inclusion. Mixed model analysis was performed to assess the difference in different domains of QoL between patients with good versus poor self-reported cosmetic satisfaction over time after adjustment for potential confounders. Depression scores were collected by means of the HADS-NL questionnaire. Chi-square test or Fisher's exact test was used to assess the difference in proportions of HADS score ≥ 8, indicating increased depression risk, between satisfied and dissatisfied patients.

**Results:**

808 patients were selected for analysis. Respectively one, two, and three years after surgery, 8% (63/808), 7% (45/626), and 8% (31/409) of patients were dissatisfied with their cosmetic outcome. Poor patient-reported cosmetic satisfaction was independently associated with impaired QoL, body image, and lower emotional and social functioning. Scores ≥ 8 on the HADS depression subscale were significantly more common in dissatisfied patients.

**Conclusions:**

Dissatisfaction with cosmetic outcome was low after breast cancer surgery followed by radiation therapy during 3 years follow-up. Knowing the association between dissatisfaction with cosmetic outcome and QoL and depression could help to improve the preoperative counseling of breast cancer patients.

## Introduction

Due to the rising incidence of breast cancer, and the improved survival rates, the number of women living with the consequences of breast cancer and breast cancer treatment is growing [1]. As a result, cosmetic satisfaction and quality of life (QoL) after breast cancer treatment are increasingly being recognized as important.

Since the introduction of breast cancer screening programs, breast cancer is often detected at an earlier stage [2]. Consequentially, the majority of breast cancer patients can be treated with breast-conserving therapy, a combination of breast-conserving surgery and breast irradiation [3, 4]. Concurrently, the interest in oncoplastic and reconstructive surgery is rising, leading to improved cosmetic results and consequently higher expectations in patients. However, the long-term degree of self-reported cosmetic satisfaction with modern treatments and how this affects QoL is yet unclear.

The aim of this study was to evaluate the prevalence of poor patient-reported cosmetic satisfaction up to 3 years following breast cancer treatment, to assess the determinants associated with poor cosmetic outcome, and to evaluate the association of poor cosmetic satisfaction with social functioning, emotional functioning, body image, and depression.

## Methods

This study was conducted within the UMBRELLA cohort (Utrecht cohort for Multiple BREast cancer intervention studies and Long-term evaLuAtion) [5]. This prospective observational cohort includes breast cancer patients referred for post-operative radiation therapy to the department of Radiation Oncology at the Utrecht Medical Center Utrecht (UMC), the Netherlands. Here, prior to the start of radiation therapy, all breast cancer patients are invited to participate in the UMBRELLA. Inclusion criteria are invasive breast cancer or ductal carcinoma in situ (DCIS), age over 18 years, and good understanding of the Dutch language. The UMBRELLA study complies with the Dutch law on Medical Research in Humans and was approved by the Medical Ethical Committee of the UMC.

Upon inclusion, all patients were asked for informed consent for the collection of clinical data and patient-reported outcomes (PROs). Clinical data were obtained through the Netherlands Comprehensive Cancer Organization (IKNL) [1]. Data on PROs were collected through self-reported questionnaires, which were collected before the start of radiation therapy (baseline) and at 3, 6, 12, 18, and 24 months after inclusion.

All patients enrolled in UMBRELLA between October 2013 and June 2018 were eligible for this study. Patients were selected when they completed the cosmetic evaluation questionnaire at 12 months after inclusion. In the cosmetic evaluation questionnaire the treated breast is compared to contralateral breast, therefore patients with mastectomy without breast reconstruction were excluded.

Cosmetic satisfaction was measured by means of a structured questionnaire by Sneeuw et al. [6]. This questionnaire was specifically designed to measure satisfaction with the breast after radiation therapy. Patients reported their satisfaction with cosmetic outcome in comparison to the contralateral breast on a 5-point Likert scale. UMBRELLA participants filled out this questionnaire at 12 months after inclusion, as scars will have matured at this time point, and again at 24 months and 36 months.

Subdomains on quality of life, emotional functioning, and social functioning were collected with the European Organization for Research and Treatment of Cancer (EORTC) QLQ-C30 questionnaires and body image was collected by means of the breast cancer specific BR23 questionnaire [7]. Each subscale contains a different number of items to be scored, and each individual item was scored on a 4-point Likert scale (body image, emotional functioning, and social functioning) or 7-point Likert scale (global QoL). The scores for global QoL, body image, emotional functioning, and social functioning were calculated using the EORTC scoring manual. Total score of one subscale ranges from 1 to 100. A higher score indicates a better outcome.

PROs on depression were collected through the Hospital Anxiety and Depression Scale (HADS, Dutch translation) questionnaire [8]. HADS is a 14-item self-rating scale with seven questions to measure the symptoms of depression. Each question has four answer options, leading to a score between 0 and 3 for each question. Higher scores indicate a higher risk of depression. An increased risk of depressive disorders was defined as a HADS score ≥ 8. A Dutch reference population (*n* = 904), matched for age and gender, was used for the EORTC QLQ-C30 and HADS depression scores [9].

Clinical data including type of surgery, axillary treatment, tumor size, radiation therapy, and primary (neoadjuvant) or post-operative systemic treatment with hormonal therapy or chemotherapy were collected through the IKNL. Information on age, height, weight and smoking behavior, was collected within the UMBRELLA cohort through a bi-annual questionnaire. Age is defined as age upon inclusion. Smoking was classified as ‘yes’ when patients were active smokers during follow-up and ‘no’ for non-active smokers. Body mass index (BMI) scores were based on mean height (m) and weight (kg) during follow-up. BMI was calculated as weight/height^2^.

### Statistics

Patient demographics and tumor and treatment characteristics were used to compare proportions, frequencies, and means with standard deviations between three groups of patients: satisfied, neutral, or dissatisfied with cosmetic outcome. Patient, treatment, and tumor characteristics of patients who responded to the cosmetic questionnaire were compared to those of patients who did not respond to the cosmetic questionnaire.

Changes in QoL, body image, emotional functioning, and social functioning between satisfied and dissatisfied patients were analyzed using a linear mixed-effect model to account for correlation between subjects over time. For mixed model analysis the self-reported cosmetic outcome was dichotomized into satisfied/neutral with cosmetic outcome and dissatisfied with cosmetic outcome. The model included a random intercept, a linear time effect, and time–cosmetic outcome interaction. We adjusted for potential confounders, i.e., age (continuous), type of surgery (lumpectomy vs. breast reconstruction), hormonal therapy, chemotherapy ± immunotherapy, BMI (≤ 25 vs. > 25), active smoking during follow-up, axillary treatment (sentinel node procedure vs. axillary lymph node procedure), radiation therapy (local vs. locoregional), and radiation therapy boost on the tumor bed. An autoregressive covariance structure of the first order was included, since it was assumed that measurements closer together in time were more correlated than measurements further apart [10].

Differences in proportions of high depression scores (a HADS-NL score ≥ 8) were compared between satisfied and dissatisfied patients, using a Chi -square test and Fisher’s exact test. A *p*- value < 0.05 was considered significant. Analyses were performed with Statistical Package for Social Sciences software (IBM SPSS Statistics version 23).

## Results

**Fig. 1:**
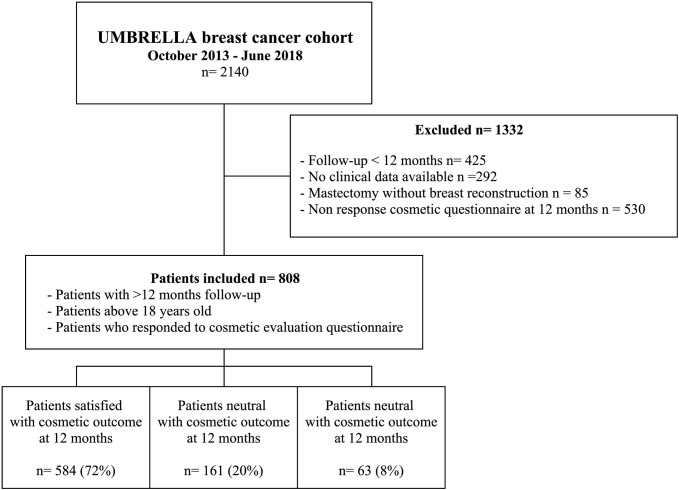
Flowchart of inclusion selected patients included in the UMBRELLA study

Between October 2013 and June 2018, 2140 patients were enrolled in the UMBRELLA cohort. Of those, 425 patients had a follow-up < 12 months, 292 patients had no clinical data available, 85 patients were treated with mastectomy without reconstruction, and 530 patients did not respond to the cosmetic questionnaire at 12 months (i.e. non-responders). These patients were excluded, resulting in 808 patients eligible for the present study (Fig. 1). Breast cancer treatment of women who responded to cosmetic questionnaires was comparable to that of patients who did not respond to cosmetic questionnaire (Table 1). There were more missing data on QoL, smoking, and BMI in non-responders in comparison to patients included in this study (respectively 41% vs. 10% and 55% vs 7%).

**Table 1:** Patient and treatment characteristics of patients who responded to the cosmetic questionnaire versus non-responders to cosmetic questionnaire

	Included in study *n* = 808	Non-responders *n* = 530
Age [mean (SD)]	58 (10)	57 (12)
Tumor size in mm [mean(SD)]	15 (12)	16 (13)
Unknown [*n* (%)]	51 (6)	21 (4)
Type of surgery		
Breast conserving surgery	769 (95)	493 (93)
Mastectomy combined with breast reconstruction	39 (5)	37 (7)
Axillary treatment^a^		
Axillary lymph node dissection	67 (8)	44 (8)
Sentinel node procedure	682 (84)	437 (82)
Unknown	59 (7)	49 (9)
Chemotherapy^b^		
Yes	278 (34)	191 (36)
No	530 (66)	339 (64)
Hormonal treatment^b^		
Yes	372 (46)	249 (47)
No	436 (54)	281 (53)
Type of radiation therapy		
Local	720 (89)	408 (77)
Locoregional^c^	74 (9)	117 (22)
Unknown	14 (2)	5 (1)
Radiation therapy boost^a^		
Yes	384 (48)	263 (50)
No	410 (51)	264 (50)
Unknown	14 (2)	3 (1)
Smoking		
Yes	84 (10)	42 (8)
No	669 (83)	196 (37)
Unknown	55 (7)	292 (55)
Body mass index^a,d^		
BMI ≤ 25	343 (43)	106 (20)
BMI > 25	410 (51)	132 (25)
Unknown/not reported	55 (7)	292 (55)
Quality of life at enrolment [mean (SD)]^e^	74 (18)	73 (18)
Unknown [*n* (%)]	79 (10)	217 (41)

Unless stated otherwise, numbers are shown as *n* (%)

^a^Total percentage other than 100% due to rounding

^b^Both primary systemic treatment and post-operative systemic treatment

^c^Including radiation therapy on periclavicular and/or axillary lymph nodes

^d^ Calculated as weight/height^2^

^e^Assessed by EORTC QLQ-C30 questionnaires, a higher score indicates better quality of life (range 0-100)

At 12 months, the proportion of satisfied, neutral, and dissatisfied patients was 72% (*n* = 584), 20% (*n* = 161), and 8% (*n* = 63), respectively (Table 2). The proportion of satisfied, neutral, and dissatisfied patients remained approximately stable over time. This proportion of dissatisfied patients was 8% (63/808), 7% (45/626), and 8% (31/409) after 1, 2, and 3 years of follow-up respectively (Table 3). Of the patients dissatisfied with cosmetic outcome at 24 months, 60% (27/45) was also dissatisfied at 12 months, whereas 40% (18/45) of patients who where previously satisfied with cosmetic outcome now were dissatisfied. At 36 months, 55% (17/31) patients were dissatisfied both at 12 months as well as at 36 months after inclusion.

**Table 2:** Baseline table: patient demographics, tumor specifics, and treatment specifics in relation to patient (dis)satisfaction with cosmetic outcome

	Satisfaction with cosmetic outcome^a^		
	Satisfied *n* = 584	Neutral *n* = 161	Dissatisfied *n* = 63
Age [mean (SD)]	58 (10)	56 (10)	56 (10)
Tumor size in mm [mean (SD)]	14 (10)	16 (10)	19 (20)
Unknown [*n* (%)]	38 (7)	11 (7)	4 (6)
Type of surgery			
Breast conserving surgery	569 (97)	147 (91)	53 (84)
Mastectomy combined with breast reconstruction^b^	15 (3)	14 (9)	10 (16)
Axillary treatment			
Axillary lymph node dissection	38 (7)	19 (11)	10 (16)
Sentinel node procedure	499 (85)	130 (81)	53 (84)
Unknown	47 (8)	12 (7)	0 (0)
Chemotherapy^c,d^			
Yes	174 (30)	72 (45)	32 (51)
No	410 (70)	89 (55)	31 (50)
Hormonal treatment^d^			
Yes	252 (43)	86 (53)	34 (54)
No	332 (57)	75 (47)	29 (46)
Type of radiation therapy			
Local	538 (92)	131 (81)	51 (81)
Locoregional^e^	42 (7)	22 (14)	10 (16)
Unknown	4 (1)	8 (5)	2 (3)
Radiation therapy boost			
Yes	276 (47)	78 (48)	30 (48)
No	304 (52)	75 (47)	31 (49)
Unknown	4 (1)	8 (5)	2 (3)
Smoking^c^			
Yes	58 (10)	20 (12)	6 (10)
No	487 (83)	130 (81)	52 (83)
Unknown	39 (7)	11 (7)	5 (8)
Body mass index^f^			
BMI ≤ 25	251 (43)	72 (45)	20 (32)
BMI > 25	294 (50)	78 (48)	38 (60)
Unknown	39 (7)	11 (7)	5 (8)

Unless stated otherwise, numbers are shown as *n* (%)

^a^Defined as satisfaction with cosmetic outcome 12 months after inclusion

^b^All breast reconstructions were performed directly after mastectomy

^c^Total percentage other than 100% due to rounding

^d^Both primary systemic treatment and post-operative systemic treatment

^e^Including radiation therapy on periclavicular and/or axillary lymph nodes

^f^Calculated as weight/height^2^

**Table 3:** Proportion of patients satisfied, neutral, and dissatisfied with cosmetic outcome during 3 years follow-up

	Satisfaction with cosmetic outcome		
	Satisfied (%)	Neutral (%)	Dissatisfied (%)
1 year follow-up	584 (72)	161 (20)	63 (8)
2 years follow-up	475 (76)	106 (17)	45 (7)
3 years follow-up	305 (75)	73 (9)	31 (8)

The respective mean age at inclusion of satisfied, neutral, and dissatisfied patients was 58, 56, and 56 years. Of the patients satisfied at 12 months after inclusion, 97% (569/584) was treated with breast-conserving surgery. This was respectively 91% of the neutral patients (147/161) and 84% of dissatisfied patients (53/63). A larger proportion of dissatisfied patients was treated with chemotherapy in contrast to neutral and satisfied patients (respectively 51%, 45%, and 30%) as well as hormonal therapy (respectively 54%, 53%, and 43%). Also, 16% of the dissatisfied patients was treated with locoregional radiation therapy in contrast to 14% of the neutral and 7% of the satisfied patients. The proportion of satisfied, neutral, and dissatisfied patients was approximately equally distributed for radiation therapy boost, active smoking, and body mass index (respectively 49–52%, 10–12% and 48-60%).

**Fig. 2:**
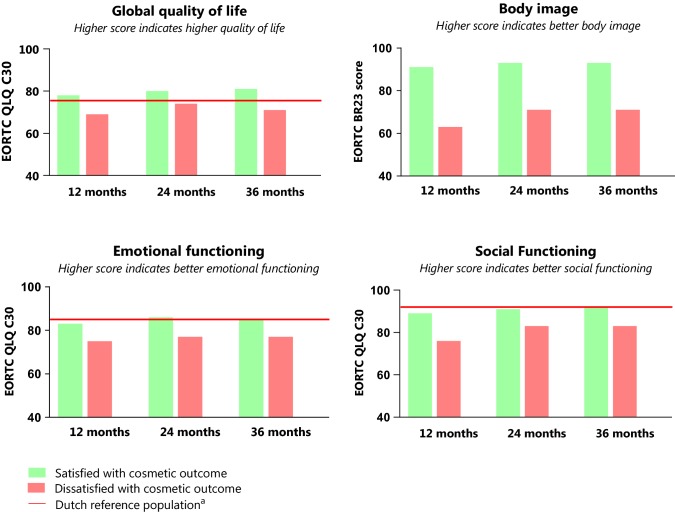
The crude levels of different domains of QoL in respect to cosmetic outcome and a matched Dutch reference non-cancer population^a^. ^a^A reference population for quality of life, emotional functioning, and social functioning was available. There is currently no reference population available for body image. Reference population included 907 women without breast cancer with comparable age to study population

Dissatisfied patients had lower levels of unadjusted QoL, body image, and social and emotional functioning compared to patients satisfied with cosmetic outcome (Fig. 2). Satisfied patients reported higher scores of global QoL in comparison to the reference population at all time-points. Social and emotional functioning of satisfied patients was comparable to that of the Dutch reference population. In contrast, dissatisfied patients reported poorer social and emotional functioning compared to the Dutch reference population during follow-up.

**Fig. 3:**
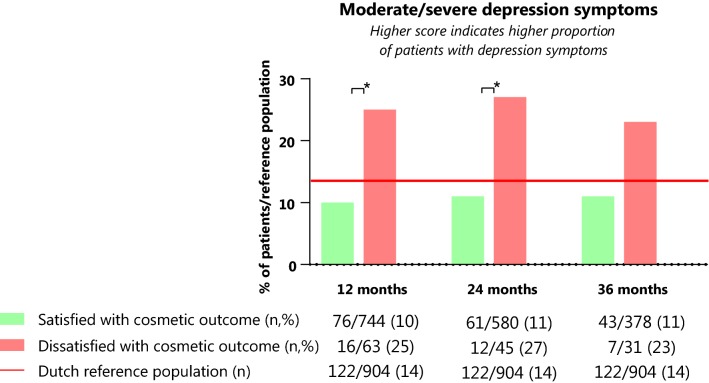
Proportion of dissatisfied and satisfied patients with a HADS-NL depression score ≥ 8 after 12, 24 , and 36 months of follow-up. *Significant difference of *p*-value < 0.05 based on Chi-square test at 12 and 24 months and Fisher's exact test after 36 months of follow-up

After adjustment for potential confounders (i.e., age, type of surgery, chemotherapy, hormonal therapy, BMI, smoking, axillary treatment, and radiation therapy ± boost), dissatisfied patients reported significantly lower QoL at 12 and 36 months (mean difference (MD) 6.9, 95% CI 2.1–11.6 and MD 8.8, 95% CI 2.7–15.0) (Table 4). After 24 months, QoL was lower in dissatisfied than satisfied patients, however this difference was not significant (MD 3.4, 95% CI − 1.9–8.7). Dissatisfied patients had a worse body image compared to satisfied patients at 12, 24, and 36 months (MD 22.2, 95% CI 18.1–26.4; MD 20.6, 95% CI 16.0–25.1; and MD 20.3, 95% CI 15.6–25.0) (Table 4). Also, dissatisfied patients reported lower emotional functioning at 12, 24, and 36 months in comparison to satisfied patients (respectively MD 6.8, 95% CI 1.7–12.0; MD 7.9, 95% CI 2.3–13.5; and MD 8.2, 95% CI 1.2–15.3). Social functioning of dissatisfied patients in comparison to satisfied patients was significantly lower at 12 and 36 months (MD 8.4, 95% CI 3.3–13.5; MD 6.3, 95% CI 0.1–12.2). Although not statistically significant, social functioning of dissatisfied patients was lower in comparison to satisfied patients at 24 months (MD 5.4, 95% CI − 0.3–10.8). There was a significantly lower proportion of patients with symptoms suggestive of possible depression (i.e., with a HADS-NL score ≥ 8) among satisfied patients in comparison to dissatisfied patients after 12 and 24 months of follow-up (respectively 10% vs. 25% and 11% vs. 27%), in contrast to 14% in the Dutch reference population (Fig. 3). This was respectively 11% and 23% after 36 months of follow-up (not statistically significant).

**Table 4:** Results of mixed model analysis with patient-reported outcome scores for patients satisfied and dissatisfied with cosmetic outcome after 12 months, 24 months, and 36 months

	12 months				24 months			36 months		
	Group	Between group difference			Between group difference			Between group difference		
		Mean	MD	95% CI	Mean	MD	95% CI	Mean	MD	95% CI
QoL	Satisfied	78.2	Ref. group		79.6	Ref. group		80.9	Ref. group	
	Dissatisfied	71.3	6.9*	2.1–11.6	76.2	3.4	− 1.9–8.7	72.1	8.8*	2.7–15.0
Body image	Satisfied	91.1	Ref. group		92.3	Ref. group		93.0	Ref. group	
	Dissatisfied	68.9	22.2*	18.1–26.4	71.7	20.6*	16.0–25.1	72.7	20.3*	15.6–25.0
Emotional	Satisfied	83.6	Ref. group		85.7	Ref. group		85.1	Ref. group	
	Dissatisfied	76.8	6.8*	1.7–12.0	77.8	7.9*	2.3–13.5	76.9	8.2*	1.2–15.3
Social	Satisfied	88.5	Ref. group		90.8	Ref. group		91.5	Ref. group	
	Dissatisfied	80.0	8.4*	3.3–13.5	85.5	5.4	− 0.3–10.8	85.2	6.3*	0.1–12.2

EORTC QLQ C30 and BR23 range from 0 to 100. A higher score indicates a better outcome. Mean scores were adjusted for age, type of surgery, chemotherapy, hormonal therapy, BMI, smoking, axillary treatment, radiation therapy, and boost. MD: mean difference, i.e., difference in mean scores between patients dissatisfied and satisfied with cosmetic outcome

*95% CI* 95% confidence interval, *Ref. group* reference group

*Significant difference with a *p*-value < 0.05

## Discussion

This prospective observational study showed that a stable proportion of 7–8% of breast cancer patients treated with breast-conserving treatment or mastectomy with immediate reconstruction were dissatisfied with respect to their cosmetic outcome up until 3 years after breast cancer treatment. Cosmetic dissatisfaction was independently associated with poorer global quality of life, body image, social functioning, and emotional functioning and a higher proportion of patients with moderate/severe depression scores.

Several other studies have described patient-reported cosmetic outcome after breast cancer treatment. In these studies, 8–20% of the patients were dissatisfied with their cosmetic outcome [11–13]. In the prospective trial of Garsa et al., 151 early-stage breast cancer patients treated with breast-conserving surgery and partial breast radiation therapy were included. The percentage of patients reporting excellent/good cosmetic outcome after respectively 3 months, 2, and 3 years after radiation therapy was 91%, 87%, and 92% [11]. Garsa et al. defined patient-reported cosmetic outcome as a combination of factors (i.e., breast size, nipple/areola location and shape, appearance of the surgical scar, breast shape, and skin color). In the older prospective study of Matory et al., 57 patients were treated with, as they described, partial mastectomy. Most patients were also treated with radiation therapy [12]. Cosmetic outcome was assessed by physical and photographic examination using a 4-point Likert scale with “no difference from contralateral breast,” “minimal difference from contralateral breast,” “moderate asymmetry,” and “gross distortion or asymmetry” representing respectively excellent, good, fair, and poor results. A good or excellent cosmetic result was reported by 80% (*n* = 50/57) of the patients after a median follow-up of 36 months, which was lower than the 93% which we found 3 years after treatment. This could be due to the fact that patients were treated with partial mastectomy. During a partial mastectomy larger tissue volume is removed in comparison to the breast-conserving surgery which is mostly performed nowadays. This can affect cosmetic outcome [14].

In accordance with the literature, we observed that younger patients were more likely to be dissatisfied with cosmetic outcome [15–18]. This might be because younger patients are more likely to receive mastectomy followed by reconstruction, resulting in a greater risk of dissatisfaction [15]. Another explanation may be that younger women are more demanding and sensitive regarding their physical appearance [16–18]. The use of tamoxifen is associated with the development of fibrosis, which might induce poorer cosmetic outcome [19, 20]. In the present study, hormonal treatment was observed more frequently in patients dissatisfied with cosmetic outcome than satisfied patients with cosmetic outcome. However, no distinction between the type of hormonal therapy was made, since data on type of hormonal therapy was often unknown. We found that axillary lymph node dissection impacted the cosmetic outcome which has been described previously. It is known that extensive axillary treatment like axillary lymph node dissection can be associated with the risk of developing lymphedema and therefore can influence the healing of the breast tissue after surgery and radiation therapy [21–23]. Results of this study also indicated that more extensive radiation therapy (i.e., locoregional radiation therapy) and type of surgery (i.e., mastectomy with immediate reconstruction) impair cosmetic outcome. Other studies showed that satisfaction with cosmetic outcome in patients treated with breast-conserving therapy depends on the amount of tissue excised during surgery, with a larger amount of tissue excised resulting in a lower level of satisfaction [14, 19, 24–27]. The higher proportion of dissatisfied patients in comparison to the neutral and satisfied patients treated with locoregional radiation therapy could be explained by the increased risk of fibrosis due to radiation therapy of the breast tissue even years after the start of radiation therapy, as the administration of an additional radiation therapy boost was distributed equally in local and locoregional treated patients [19, 28–32]. In the “boost vs. no boost” trial, 5318 early-stage breast cancer patients were randomized to additional boost on the tumor bed or no further treatment [19]. An independent association between radiation therapy boost and poorer cosmetic outcome was seen after 3 years follow-up. In our study, the proportion of patients treated with radiation therapy boost was approximately equally distributed amongst the satisfied, neutral, and satisfied patients. However, this was only evaluated at 1 year follow-up. Breast and chest wall fibrosis develop over the course of many years and our cohort may not be mature enough to assess the impact of radiation therapy boost. In contrast to other studies, smoking and BMI had no influence on cosmetic outcome in the present study.

We aimed to assess the impact of cosmetic (dis)satisfaction on the different domains of quality of life and found a strong association between the two. Since self-reported cosmetic outcome and quality of life and depression scores were measured simultaneously, we do not know the direction of the association. It could be that dissatisfaction with cosmetic outcome causes higher depression scores and lower quality of life. However, the contrary—higher depression scores or lower quality of life causing dissatisfaction with cosmetic outcome—may also be the case.

Previously, only the impact of cosmetic outcome on global quality of life or body image was assessed. Hau et al. evaluated the association between global quality of life and cosmetic outcome in 688 breast cancer patients treated with post-operative radiation therapy after breast-conserving surgery, using the EORTC QLQ-C30 [33]. Patient-reported cosmetic outcome was dichotomized into good/excellent vs. fair/poor. Prior to radiation therapy, at 5 and 10 years follow-up, patients dissatisfied with cosmetic outcome reported a significantly lower global QoL score than satisfied patients (differences of 6.3, 9.6, and 7.3 points respectively on EORTC QLQ-C30). These results are comparable with our results.

After adjustment for patient and treatment related factors, patients satisfied with cosmetic outcome had similar emotional and social functioning in comparison to a Dutch reference non-cancer, female population during 3 years follow-up. Also, a smaller proportion of satisfied patients reported higher HADS scores in comparison to the Dutch reference population. Dissatisfied patients however, scored worse on all domains. Dissatisfaction with cosmetic appearance could be influenced by expectations of the cosmetic result after surgery [34, 35]. Therefore, managing patients’ expectations and providing information about cosmetic results of patients with similar characteristics and expectations seems important, possibly by early referral to a plastic surgeon.

Our study suffers from some limitations. There were 530 patients who did not respond to the cosmetic questionnaire. Even though there were no differences in patient and treatment characteristics, we may have over- or underestimated the proportion of dissatisfied participants. Also, we cannot rule out that the association between cosmetic outcome and quality of life was distorted: it may, for example, have been stronger, when cosmetically dissatisfied women with (very) low quality of life scores were more likely to be non-responders. In the present study, smoking was defined as active smoking during follow-up. Information on the number of pack years and the start date of smoking was not available. Therefore, the impact these factors could have had on the cosmetic outcome, could not be taken into account. Breast size prior to surgery, post-operative complications such as infection and seroma, and tumor localization within the breast are known to be risk factors for poor cosmetic outcome [13, 15, 22, 36]. These patient characteristics were not collected within the cohort. Also, the cosmetic evaluation questionnaire was only sent to patients 12, 24, and 36 months after inclusion. Consequently, we miss information on the satisfaction with cosmetic outcome shortly after surgery or prior to breast cancer treatment.

Nonetheless, this study provides insights into the longitudinal patient satisfaction with cosmetics after breast cancer and breast cancer treatment. Outcomes from this study emphasizes the importance of post-treatment care of breast cancer patients and shared decision making prior to breast cancer treatment.

## Conclusion

In conclusion, dissatisfaction with cosmetic outcome in the first 3 years after breast surgery and post-operative radiation therapy is low, i.e., 7–8%. As cosmetic outcome was associated with reduced quality of life, poorer body image, reduced social and emotional functioning, and increased depressive symptom scores, counseling on the impact of satisfaction with cosmetic outcome on the quality of life could be considered.

## Abbreviations

BMI: Body mass index
DCIS: Ductal carcinoma in situ
EORTC: European Organization for Research and Treatment of Cancer
HADS: Hospital Anxiety and Depression Scale
IKNL: The Netherlands Comprehensive Cancer Organization
PROs: Patient reported outcome(s)
QoL: Quality of life
UMBRELLA: Utrecht cohort for Multiple BREast cancer intervention studies and Long-term evaLuAtion
